# Effect of papaya (*Carica papaya*) leaf extract as dietary growth promoter supplement in red hybrid tilapia (*Oreochromis mossambicus* × *Oreochromis niloticus*) diet

**DOI:** 10.1016/j.sjbs.2022.03.004

**Published:** 2022-03-09

**Authors:** Noor Khalidah Abdul Hamid, Peace Onas Somdare, Khadijah Abdullah Md Harashid, Nurul Ain Othman, Zulhisyam Abdul Kari, Lee Seong Wei, Mahmoud A.O. Dawood

**Affiliations:** a School of Biological Sciences, Universiti Sains Malaysia, 11800 Pulau Pinang, Malaysia; b Department of Zoology, Federal University Lokoja, P.M.B. 1154 Lokoja, Nigeria; c Department of Agricultural Science, Faculty of Agro-Based Industry, Universiti Malaysia Kelantan, 17600 Jeli, Kelantan, Malaysia; d Department of Animal Production, Faculty of Agriculture, Kafrelsheikh University, 33516 Kafrelsheikh, Egypt; e The Center for Applied Research on the Environment and Sustainability, The American University in Cairo, 11835 Cairo, Egypt

**Keywords:** Papaya leaf extract, Growth promoter, Feed additive, Red hybrid tilapia, Growth condition

## Abstract

The purpose of this experiment was to examine the potential use of *Carica papaya* leaf extract as a supplement to promote growth and improve feed utilization in red hybrid tilapia. Five diets were formulated containing isolipidic (80 g/kg) and isonitrogenic (350 g/kg) levels. All feeds contained similar types and amounts of raw materials but differed in the inclusion of papaya leaf extract (0, 5, 10, 20 and 40 g/kg feed). The initial size of fish used was 2.3 ± 0.01 g. Each diet was performed in triplicate tanks, and the feeding period was 12 weeks. Fish fed diet containing 2% papaya leaf extract (PLE) had the highest final weight, 31.14 ± 1.47 g, followed by 1% PLE (27.27 ± 1.75 g). These two diets (1% and 2%) were also showed significant improvements of weight gain, SGR, and feed efficiency of the red hybrid tilapia (p < 0.05). However, papaya leaf extract did not affect the HSI, VSI, PER, digestive enzymes activity, blood composition, and survival rate. Supplementing the diets with papaya leaf extract lowered serum urea. Findings of this research suggest that adding papaya leaf extract to the diet of red hybrid tilapia improves growth and feed efficiency without adversely affecting blood parameters. Therefore, an inclusion level between 1% and 2% of the papaya leaf extract is recommended as a feed additive to promote red hybrid tilapia fry growth.

## 1 Introduction

Tilapia is cultured globally for a cheap, high-quality protein source (Canonico et al., 2005) and is Malaysia's second most farmed freshwater species, after African catfish, *Clarias gariepinus* (Mohamad et al. 2021). Like most developing countries, the vast majority of the aquaculture operators in Malaysia are small-scale farmers with limited capital, technologies, and space. One of the reasons that tilapia has become a popular species for small-scale operation is because the operational cost is reasonable to these farmers, especially the feed cost (Mohammadi et al., 2022a, Mohammadi et al., 2022b). Tilapia is an omnivorous fish that tolerates diverse food resources with lower protein requirements than carnivorous fish (Ringø, 2020). Red hybrid tilapia generally requires between 25% and 35% of protein; thus, the feed cost is cheaper (El-Sayed et al., 2004, Twibell and Brown, 1998). However, during the lockdowns, the price of commercial feed spiked, increasing daily operational costs. For the small-scale farmers, the price spikes negatively impact their revenue. Therefore, reducing the growing period of the fish by promoting better growth may be beneficial to the aquaculture industry, especially the small-scale farmers.

Dietary supplements are incorporated into aquatic feed to help the body systems process and absorb the nutrients they get from the food they ingest (Dawood et al., 2021, Zheng et al., 2020, Alemayehu et al., 2018) and to improve fish health (Shekarabi et al., 2022). Exogenous enzymes, amino acids, and effective microbes are just some of the feed additives that can help fish grow (Kari et al., 2022, Mohammadi et al., 2022a, Mohammadi et al., 2022b, Kari et al., 2021, Cerqueira et al., 2020, Encarnação, 2016, Castillo and Gatlin, 2015). Proteolytic enzymes are increasingly being used in fish feed as additives, in part due to the rapid expansion of the aquaculture industry and safer approach to reduce the operational cost. Exogenous proteolytic enzymes may be derived from any living organism, including animals, plants, and microorganisms (Zulhisyam et al., 2020, Agyei et al., 2019, Singh et al., 2019, Walsh et al., 1993). Appropriate incorporation of exogenous enzymes into the feed will compensate for endogenous enzyme deficiency and boost growth performance (Zheng et al., 2020, Encarnação, 2016).

Papaya plant (*Carica papaya*) produces a specific proteolytic enzyme called papain. This botanically derived exogenous enzyme has numerous health benefits, especially as a protein-dissolving substance (Isa, 2010). Previous studies showed that papain inclusion in aquafeed promotes growth performance and improved feed utilisation in sterlet (*Acipencer ruthenus*; Wiszniewski et al., 2022), African catfish (*Clarias gariepinus*; Rachmawati et al., 2020), mahseer (*Tor tambra*; Muchlisin et al., 2016) and rohu (*Labeo rohita*; Khati et al., 2015). Papain is predominantly the plant's latex, but the enzyme is also detected in every part of the plant (Mamboya and Amri, 2012). Papaya leaf is often regarded as agricultural waste. Nevertheless, it may have the potential to be utilised as a sustainable fish growth promoter in aquafeed due to the presence of papain (Vij and Prashar, 2015, Mahanty et al., 2013, Isa, 2010). Previously, the use of papaya leaf extract in fish mainly focused on its microbicidal properties against fish pathogens (Azizah and Fasya, 2019, Fakoya et al., 2019), and there have been limited reports on the potential of papaya leaf in aquafeed as a growth supplement. Thus, this study aims to investigate the effect of papaya leaf extract on growth, feed utilisation, the activity of digestive enzymes, haematology, and blood biochemistry components of red hybrid tilapia.

## 2 Material and methods

### 2.1 Ethics statements

This study followed the National Institutes of Health's guide for the care and use of laboratory animals and was approved by the Universiti Sains Malaysia's Animal Ethical Committee. (Ref: USM/IACUC/2021/(127)1128).

### 2.2 Experimental fish and husbandry conditions

The red hybrid tilapia (*Oreochromis mossambicus* × *Oreochromis niloticus*) fingerlings were purchased from Aquatech Sdn. Bhd. and transported to the Kompleks Penyelidikan Akuatik (L24), Universiti Sains Malaysia. Two weeks prior to the experiment, the fish were acclimatised to ambient laboratory conditions upon arrival. During the acclimatisation phase, fish were provided with a commercial tilapia pellet containing 35% protein and 8% lipid twice a day.

Healthy fish weighing an average of 2.3 g were randomly assigned to fifteen glass aquariums (70 cm length × 46.7 cm height × 30.0 cm width) with all the water levels were set at 30 cm height. Each aquarium was supplied with ten fish. All tanks had continuous aeration, and the fish were cultured in carbon-filtered water. The water in the culturing tank was changed on a daily basis. The light cycle of the indoor system used for this experiment was set to 12 h. The feeding trial was performed in 12 weeks in which the fish were fed to satiety with the formulated diets twice daily at 09:00 and 17: 00 h. Each diet was replicated in three tanks. Every two weeks, growth progress was tracked by weighing the fish in bulk according to the tank. The fish were food-deprived for 24 h before being handled to alleviate stress. Food intake was measured daily. The fish were weighed individually during final sampling to determine the final weight.

During the study, temperature, pH, and DO of culture condition were maintained at 25.84 ± 0.07 °C, 6.70 ± 0.06 and 7.2 ± 0.09 mg/L, respectively. Meanwhile, the level of dissolved properties such as total ammonia nitrogen (0.84–1.06 mg/L), nitrate (0.83–1.05 mg/L), nitrite (0.07–0.11 mg/L), phosphorus (∼0.70 mg/L) and was maintained within the acceptable range throughout the study.

### 2.3 Experimental design and feed preparation

A feeding trial was performed to assess the potential application of papaya leaf extract in aquafeed for tilapia. Five practical diets comprising the same protein level (35%), lipid (8%), and energy were prepared. These diets were prepared to contain a different level of papaya leaf extract (Bionutricia) in range of 0%, 0.5%, 1.0%, 2%, and 4%. These experimental diets incorporated a high level of plant protein such as soybean meal and Azolla meal, and an insect meal, Hermetia larvae meal, to reduce the fish meal usage. Table 1 shows the formulation and chemical analysis of the experimental diets.

**Table 1:** Diet formulations and proximate composition of experimental feed.

Ingredients (g/100)	Experimental diets containing a different level of papaya leaf extract				
	0.0%	0.5%	1.0%	2.0%	4.0%
Fish meal^1^	11.30	11.30	11.30	11.30	11.30
Soybean meal^2^	34.50	34.50	34.50	34.50	34.50
Corn starch^3^	14.25	14.25	14.25	14.25	14.25
Hermetia larvae meal	8.95	8.95	8.95	8.95	8.95
Azolla	22.50	22.50	22.50	22.50	22.50
Palm oil^4^	3.50	3.50	3.50	3.50	3.50
Vitamin premix^5^	0.40	0.40	0.40	0.40	0.40
Mineral premix^6^	0.10	0.10	0.10	0.10	0.10
Celite^7^	0.50	0.50	0.50	0.50	0.50
Papaya leaf extracts	0.00	0.50	1.00	2.00	4.00
CMC	4.00	3.50	3.00	2.00	0.00
Proximate composition (%)					
Moisture	7.27	7.11	7.14	7.14	7.12
Protein	36.10	36.50	36.8	36.30	36.30
Lipid	7.91	7.87	8.04	7.96	8.24
Fibre	17.86	17.34	18.01	18.12	18.00
Ash	8.95	8.84	8.79	8.82	8.76
Nitrogen Free Extracts^8^	21.91	22.34	21.22	21.66	21.58
Gross Energy (kJ/100 g)	303.23	306.19	304.44	303.48	305.68

1 Danish fishmeal.

2 Soybean meal.

3 Corn starch.

4 Palm oil.

5 Vitamin premix (Rovimix 6288; F.Hoffman La-Roche Ltd, Basel, Switzerland), containing (per kg, dry weight): Vitamin A, 50 million IU; Vitamin D3, 10 million IU; Vitamin E, 130 g; Vitamin B1, 10 g; Vitamin B2, 25 g; Vitamin B6, 16 g; Vitamin B12, 100 mg; biotin,500 mg; panthothenic acid, 56 g; folic acid, 8 g; niacin, 200 g; anti-cake20 g; antioxidant, 200 mg; Vitamin K3, 10 g; and Vitamin c, 35 g.

6 Mineral premix (g/kg)-cobalt carbonate, 100 mg; copper sulphate, 780 mg; magnesium sulphate, 137 g;mmanganese oxide, 800 mg; potassium chloride, 50 g; potassium iodide, 150 mg; sodium chloride, 60 g; sodium selenite, 200 mg and zinc oxide, 1.5 g; calcium lactate, 327 g; ferrous sulphate, 25 g; calcium phosphate (monobasic), 397.5 g.

7 Celite.

8 Nitrogen free extract: 100 - (moisture + protein + lipid + ash + fibre).

### 2.4 Growth performance and body indices

The calculation for growth performances, food efficiency and body indices are as following:

Weight gain (WG) = final weight–initial weight.
Feed conversion ratio = total feed intake. weight gain^−1^.
Specific Growth Rate = (ln Wt_final_–lnWt_initial_). duration^−1^.
Protein efficiency ratio (PER) = weight gain.total protein intake^−1^.
Survival = final number fish/initial number fish × 100.
Hepatosomatic index (HSI %) = (liver weight.body weight^−1^) × 100.
Viscerosomatic Index (VSI %) = (viscera weight.body weight^−1^) × 100.

### 2.5 Proximate analysis

AOAC methods were used to determine the moisture, protein, lipid, fibre, and ash content of raw ingredients, experimental diets, and fish (AOAC, 1997).

### 2.6 Tissue collection

The fish were euthanised during the last sampling by exposing them to an overdose of Tricaine Methanesulfonate (MS-222) bath. Three fish from each tank were chosen at random to be measured for HSI and VSI values on the entire body, visceral, and liver. Another two random fish of each tank were chosen to collect their stomach and intestine tissues for enzymatic testing. Tissues were promptly dissected and placed on ice in a tube. These tissues were kept at −20 °C until they were needed.

### 2.7 Enzyme assays

A ratio of 1:3 tissue to ice-cold distilled water was used to prepare enzyme assays. To separate the supernatant and pellet, the homogenates were centrifuged at 4 °C, 30,000*g* for 30 min. The supernatant was aliquot to a new microcentrifuge tube and kept at −20 °C. The Bio-Rad protein assay used to determine the total protein content of the tissues according to the protocol.

Walter's casein-hydrolysis method (1984) used to determine protease activity. L-tyrosine served as a standard, and stomach tissue was used to measure protease activity Worthington's (1982) starch hydrolysis process was used to determine the sample's specific amylase activity, with maltose serving as the standard. Lipase activity was assayed by titration and determined by measuring using Bier's method with modifications (Bier, 1995). The reaction tube was titrated with 0.01 M sodium hydroxide until the endpoint was reached. Lipase activity was calculated using the volume of sodium hydroxide solution used to titrate the endpoint.

### 2.8 Haematological parameters

Three fish were randomly selected from each treatment. The fish were food-deprived for 24 h prior to handling. An overdose of Tricaine Methanesulfonate (MS-222) was used to euthanise the fish. Blood was withdrawn immediately from freshly euthanised fish using a 1cc sterile syringe and collected in heparinised tubes. The analysis of blood haematology and biochemistry was conducted according to Kari et al. (2021).

### 2.9 Statistical analysis

The statistical analysis of data on proximate composition, body indices, growth performance, digestive enzyme activities, and haematology was carried out using the SPSS 26 software. Statistical analysis used was one-way ANOVA, and a post-hoc test was performed when necessary.

## 3 Results

### 3.1 Growth performance

An average of 2.37 ± 0.02 g of red hybrid tilapia was used for this study (p > 0.05), as shown in Table 2. The highest final body weight from this study was recorded in tilapia fed with 2% papaya leaf extract (31.14 ± 1.47 g, p < 0.05), followed by 1% papaya leaf extract (27.27 ± 1.75; p < 0.05). Diets containing 1% and 2% papaya leaf extract also improved weight gain and SGR of the red hybrid tilapia substantially (p < 0.05). Feed consumption was found significantly higher in fish supplied with 2% papaya leaf extract in comparison to all treatments except for the 1% papaya leaf extract fish. The inclusion of 1% (1.44) and 2% papaya leaf extract (1.39) had a lower FCR value in comparison to other treatments (p < 0.05). However, the PER was not affected by the treatments. No effect of papaya leaf extract inclusion on hepatosomatic and visecerosomatic index of the fish was detected (p > 0.05). No differences in survival rate was observed between treatments (p > 0.05).

**Table 2:** Growth parameters and body indices of red hybrid tilapia fed with formulated diets for 12 weeks. Data are presented in mean ± SEM. Different superscripts in each row indicate a significant difference (p < 0.05).

Growth index	Diets				
	0.0%	0.5%	1.0%	2.0%	4.0%
IW (g)	2.39 ± 0.07	2.39 ± 0.07	2.35 ± 0.07	2.36 ± 0.08	2.39 ± 0.07
FW (g)	21.30 ± 1.22^a^	21.09 ± 1.15^a^	27.27 ± 1.75^b^	31.14 ± 1.47^c^	22.24 ± 1.16^a^
WG (g)	18.90 ± 1.17^a^	18.09 ± 1.18^a^	25.31 ± 3.30^b^	28.88 ± 1.62^b^	19.45 ± 0.22^a^
SGR (% day^−1^)	2.60 ± 0.06^a^	2.55 ± 0.07^a^	2.92 ± 0.14^b^	3.07 ± 0.05^b^	2.63 ± 0.01^a^
FCR	1.65 ± 0.13^b^	1.65 ± 0.04^b^	1.44 ± 0.02^a^	1.39 ± 0.01^a^	1.68 ± 0.03^b^
Feed intake (g)	30.79 ± 0.65^a^	29.81 ± 1.79^a^	36.41 ± 4.34^ab^	40.27 ± 1.86^b^	32.72 ± 0.98^ab^
PER	1.70 ± 0.242	1.73 ± 0.09	1.88 ± 0.04	1.97 ± 0.03	1.64 ± 0.06
VSI (%)	11.14 ± 0.63	11.45 ± 1.02	10.88 ± 0.67	10.68 ± 0.82	9.755 ± 0.68
HSI (%)	1.69 ± 0.22	1.59 ± 0.15	1.57 ± 0.22	1.83 ± 0.19	1.54 ± 0.37
Survival (%)	96.67 ± 3.33	100.00 ± 0.00	90.00 ± 5.77	93.33 ± 3.33	93.33 ± 3.33

Abbreviations: HSI-Hepatosomatic index; IW-initial weight; FW-final weight; SGR-Specific growth rate; WG-weight gain; FCR-Feed conversion ratio; VSI-Visceral somatic index.

### 3.2 Proximate composition of the whole body

Table 3 displays the results of proximate nutrient composition from whole fish. The moisture, protein, and lipid content of whole fish were not affected by papaya leaf extract inclusion (p > 0.05).

**Table 3:** Proximate composition of the whole body of red hybrid tilapia fed with different experimental feed for 12 weeks. Data are presented in mean ± SEM, n = 9 fish per treatment.

Proximate composition (%)	Initial	Diets				
		0.0%	0.5%	1.0%	2.0%	4.0%
Moisture	76.21	77.07 ± 0.50	77.16 ± 0.50	76.35 ± 1.27	76.70 ± 0.52	77.01 ± 0.79
Protein	61.40	70.60 ± 0.51	70.97 ± 0.56	71.43 ± 1.22	70.80 ± 0.31	71.23 ± 0.75
Lipid	4.32	6.67 ± 0.03	7.98 ± 0.55	7.89 ± 1.36	7.42 ± 0.56	7.21 ± 0.76
Ash	25.55	14.17 ± 0.83	14.37 ± 0.53	15.11 ± 1.30	13.93 ± 0.49	13.90 ± 0.45

### 3.3 Digestive enzymes

Table 4 shows the activity of gastrointestinal amylase, protease, and lipase in red hybrid tilapia fed with various doses of papaya leaf extract. Inclusion of papaya leaf extract up to 2% did not significantly affected the protease activity, but at 4% inclusion, papaya leaf extract in the feed altered gastric protease activity of red hybrid tilapia (p < 0.05). There were no obvious alterations in intestinal amylase of fish supplied with different levels of papaya leaf extract (p > 0.05). However, the activity of intestinal lipase was altered by the treatments in which the lipase in fish fed with 1% papaya leaf extract feed was significantly elevated than other treatments (p < 0.05).

**Table 4:** Digestive enzymes activity in the gastrointestinal tract of red hybrid tilapia after 12 weeks of feeding trial. Data are presented in mean ± SEM, n = 6 fish per treatment. Different superscripts in each row indicate a significant difference (p < 0.05).

(U/mg protein)	Diet				
	0%	0.50%	1%	2%	4%
Protease	3.370 ± 0.549^a^	3.525 ± 0.564^a^	3.425 ± 0.366^a^	2.445 ± 0.316^a^	4.865 ± 0.135^b^
Amylase	0.286 ± 0.037	0.310 ± 0.018	0.307 ± 0.076	0.333 ± 0.071	0.308 ± 0.025
Lipase	47.065 ± 6.474^a^	44.427 ± 9.836^a^	68.987 ± 3.591^b^	35.781 ± 2.328^a^	46.548 ± 4.113^a^

### 3.4 Blood biochemistry

The parameters of blood biochemistry are shown in Table 5. There were no significant differences (p < 0.05) in the levels of total protein (TP), glucose (GLU), alkaline phosphatase (ALKP), globulin (GLOB), alanine aminotransferase (ALT), creatinine (CREA), aspartate aminotransferase (AST), albumin (ALB), total bilirubin (TBIL) ALT, AST, GLU, and cholesterol (CHOL) of red hybrid tilapia fed with dietary treatments. The inclusion of papaya leaf extract to the diet altered blood urea nitrogen (BUN), so that the level of BUN decreased as the amount of papaya leaf extract inclusion increased (p < 0.05).

**Table 5:** Blood biochemistry of red hybrid tilapia fed diets containing different levels of papaya leaf extract for 12 weeks, n = 3.

Plasma index	Experimental diets				
	0.0 %	0.5 %	1.0 %	2.0 %	4.0 %
ALB (g/dl)	1.34 ± 0.26	1.56 ± 0.07	1.49 ± 0.11	1.46 ± 0.09	1.45 ± 0.08
TP (g/dl)	2.93 ± 0.17	2.84 ± 0.08	3.10 ± 0.25	3.44 ± 0.43	3.23 ± 0.32
CREA (mg/dl)	0.99 ± 0.12	0.87 ± 0.07	0.94 ± 0.05	0.86 ± 0.12	0.83 ± 0.04
BUN (mg/dl)	2.53 ± 0.07^bc^	2.63 ± 0.07^c^	2.45 ± 0.02^abc^	2.23 ± 0.13^a^	2.29 ± 0.11^ab^
ALT (u/l)	23.30 ± 0.70	22.93 ± 0.64	24.53 ± 0.64	23.57 ± 1.09	23.30 ± 0.87
ALKP (u/l)	34.93 ± 2.32	42.77 ± 9.47	45.27 ± 8.62	37.50 ± 1.91	35.63 ± 0.64
CHOL (mg/dl)	27.33 ± 3.01	25.17 ± 1.73	25.70 ± 1.48	26.13 ± 1.99	25.67 ± 1.55
CA (mg/dl)	5.69 ± 0.36	6.34 ± 0.29	6.03 ± 0.28	6.03 ± 0.28	5.50 ± 0.31

Abbreviations: ALT-Alanine aminotransferase; ALB-Albumin; AST-Aspartate aminotransferase; ALKP-Alkaline phosphatase; TBIL-Total bilirubin; CHOL-Cholesterol; CREA-Creatine; GGT-Gammaglutamyltransferase; GLU-Glucose; BUN-Blood urea nitrogen; GLOB-Globulin; TP-Total protein.

### 3.5 Blood haematology

The haematological indices of red hybrid tilapia fed with experimental diets are shown in Table 6. Papaya leaf extract showed no significant influence (p > 0.05) on white blood cells (WBC), lymphocytosis (LYM), monocytes (MON), and blood granulocytosis (GRA) of red hybrid tilapia fed. The experimental diets also had no significant effect (p > 0.05) on blood cell (RBC), haematocrit (HCT), mean corpuscular volume (MCV), mean corpuscular haemoglobin (MCH), and mean corpuscular haemoglobin concentration (MCHC). However, a significant difference in red cell distribution width (RDW) was noted in fish fed on 1% papaya leaf extract diet, with no specific trend to the changes (p < 0.05). The experimental diets showed a significant impact on platelet (PLT), procalcitonin (PCT), and platelet distribution width (PDW), in which fish fed with 1% papaya leaf extract had a higher level (p < 0.05) with no trend to the variations. No difference was observed on the mean platelet volume (MPV).

**Table 6:** Haematological parameters of red hybrid tilapia maintained with experimental diets. Data are presented in mean ± SEM, n = 3 fish per treatment. Different superscripts in each row show a significant difference (p < 0.05).

Parameters	Diets				
	0%	0.5%	1.0%	2.0%	4%
WBC (10^3^/μl)	78.07 ± 20.06	82.77 ± 17.81	86.87 ± 6.38	78.27 ± 25.35	61.57 ± 29.00
LYM	72.00 ± 17.13	74.73 ± 13.73	81.97 ± 4.29	68.47 ± 20.18	57.70 ± 26.68
MON	5.30 ± 2.92	7.03 ± 4.02	4.37 ± 2.03	8.37 ± 4.98	3.47 ± 2.49
GRA	0.80 ± 0.35	1.00 ± 0.67	0.53 ± 0.30	1.43 ± 0.96	0.40 ± 0.26
LYM (%)	93.50 ± 2.70	91.97 ± 4.25	94.67 ± 2.26	90.50 ± 5.06	95.70 ± 2.44
MON (%)	5.53 ± 2.57	7.03 ± 3.67	4.77 ± 1.97	8.03 ± 4.33	3.70 ± 4.31
GRA (%)	0.97 ± 0.26	1.00 ± 0.61	0.57 ± 0.29	1.47 ± 0.78	0.60 ± 0.15
RBC (10^3^/μl)	1.26 ± 0.16	1.38 ± 0.38	1.47 ± 0.13	1.22 ± 0.35	0.96 ± 0.39
HGB (g/dl)	7.30 ± 0.36	7.00 ± 1.40	7.57 ± 0.64	8.13 ± 0.85	6.87 ± 0.37
HCT (%)	15.17 ± 3.99	20.43 ± 5.28	16.10 ± 4.40	15.40 ± 4.80	18.95 ± 2.05
MCV (μm^3^)	115.70 ± 19.15	148.53 ± 6.31	110.07 ± 22.71	122.30 ± 6.54	140.60 ± 3.20
MCH (pg)	59.80 ± 6.80	53.17 ± 4.95	52.70 ± 0.81	82.43 ± 29.30	52.45 ± 0.35
MCHC(g/dl)	57.73 ± 18.36	35.77 ± 3.00	52.17 ± 10.55	70.73 ± 29.25	37.35 ± 1.15
RDW (%)	11.57 ± 2.84^ab^	7.77 ± 0.54^a^	24.00 ± 8.70^b^	14.4 7 ± 5.39^ab^	8.45 ± 1.15^a^
PLT (10^3^/μl)	68.00 ± 28.73^ab^	31.00 ± 9.61^a^	137.00 ± 54.08^b^	63.67 ± 39.07^ab^	35.00 ± 15.82^a^
MPV (μm^3^)	6.95 ± 0.75	6.20 ± 0.4	6.45 ± 0.75	6.75 ± 0.05	6.77 ± 0.15
PCT (%)	0.05 ± 0.04^a^	0.01 ± 0.00^a^	0.12 ± 0.011^b^	0.06 ± 0.03^ab^	0.02 ± 0.00^a^
PDW (%)	6.40 ± 0.80^a^	6.95 ± 0.05^ab^	7.80 ± 1.00^ab^	9.45 ± 1.15^b^	8.20 ± 0.70^ab^

Abbreviations: PDW-Platelet Distribution Width; MCV-Mean Corpuscular Volume; RBC-Red blood cell; MCH-Mean Corpuscular Haemoglobin; WBC-White blood cell; MON-Monocytes; MCHC-Mean Corpuscular Haemoglobin Concentration; HGB-Haemoglobin; RDW-Red Cell Distribution Width; LYM-Lymphocytosis; PCT-Procalcitonin; MPV-Mean Platelet Volume; GRA-Granulocytosis; PLT- Platelet; HCT, Hematocrit.

## 4 Discussion

The inclusion of papaya leaf extract in the red hybrid tilapia feed was aimed to determine its potential as a growth promoter supplement. In this study, 1% and 2% papaya leaf extract inclusion in the feed improved growth performance, FCR, and SGR values of red hybrid tilapia. This finding is consistent with several studies that found papaya leaf, skin, and seed extracts increased growth efficiency in *Oreochromis niloticus* (Kareem et al., 2016), *Cyprinus carpio* (Tewari et al., 2018), Heteroclarias (Anyanwu et al., 2007), *Clarias gariepinus* (Olusola & Nwokike, 2018), and *Lates calcarifer* (Ganzon-Naret, 2015). We also noted that between 1% and 2% papaya leaf extract inclusion levels, the 2% inclusion promotes better growth performance. A similar observation was also stated by Olusola and Nwokike (2018), where 2% inclusion of papaya leaf extract promoted the growth of African catfish than 1% inclusion level. Further, Kareem et al. (2015) reported 2% papaya seed extract is beneficial for Nile tilapia. It is noted that 4% papaya leaf extract did not affect growth or feed efficiency. A similar trend was observed in common carp, where feeding the fish with 2.5% papaya leaf extract helped the carp grower faster than fish maintained with 5% papaya leaf extract (Tewari et al. 2018). An improved SGR value for diets containing 1% and 2% papaya leaf extract obtained compared to the control diet may be due to papain presence in papaya leaf that may enhance red hybrid tilapia growth performance. Papain has been documented to increase growth in mahseer (Muchlisin et al., 2016), rohu (Khati et al., 2015), African catfish (Rachmawati et al., 2020), sterlet (Wiszniewski et al., 2022), Nile tilapia (Rostika et al., 2018a), carp (Tewari et al., 2018) and pangasius (Rostika et al., 2018).

On the other hand, the efficacy of papaya leaf extract on feed efficiency was not observed at 0.5% and 4% of inclusion level, implying an optimal dose effect in employing the extract in aquafeed. In this study, the formulated diets contained 17% of dietary fibre, which is considered a higher level than the recommended amount for most fish feed that is 5%. The high plant protein content is meant to reduce the inclusion level of fish meal and increase the amount of plant protein, including sustainable feed ingredients such as Azolla and Hermetia larvae meal (Hamid, 2021). We found that adding 1% and 2% papaya leaf extract improved the FCR value even at a high level of dietary fibre. This finding is consistent with the majority of the studies that have been reported. The addition of papaya extract or papain has been identified as a factor in improved feed efficiency for fish (Rachmawati et al., 2020, Kareem et al., 2016, Muchlisin et al., 2016; Goda et al., 2012; Farrag et al., 2013).

In this current study, the crude protein of the whole-body of hybrid tilapia in all treatments was unaltered, consistent with previous studies on Nile tilapia treated with papaya leaf and seed extracts (Kareem et al., 2016, Farrag et al., 2013). Nevertheless, unlike previous findings in Nile tilapia, where papaya extract or papain reduced lipid composition, the presence of papaya leaf extract showed no impact on the overall lipid profile of red hybrid tilapia in this study (Kareem et al., 2016, Farrag et al., 2013). The hepatosomatic index (HSI) was analysed as an indicator of the fish's energy reserves. It is proposed that an unaltered hepatosomatic index (HSI) in both treatments of this study, control, and papaya leaf extract, enables red hybrid tilapia to have a regular metabolic rate without tempering the energy reserved. Also, the VSI values in this study may suggest a normal gastrointestinal condition even with the introduction of papaya leaf extract to the diet.

In this study, the digestive enzymes amylase, lipase and protease were examined to observe their activities. The amylase activity of the hybrid red tilapia was far from being affected by the addition of papaya leaf extract, but the lipase and protease activities were altered due to the treatments. However, in the African catfish, the addition of papaya leaf extract decreases amylase and increases lipase, according to Rachmawati et al. (2020). In the case of gastric protease, a significant alteration was observed when the inclusion level of papaya leaf extract was at 4%. Although papain has been reported to change gastrointestinal proteases in Heteroclarias and sterlet, the modifications observed did not show a consistent pattern (Rachmawati et al., 2020; Wiszniewski et al., 2022). Lin and colleagues (2007) reported that total protease activity was elevated in the intestine of juvenile hybrid tilapia and improved weight gain when exogenous enzymes were added to the meal.

However, in this study, the 4% papaya leaf extract fed fish displayed the highest protease activity and amongst the lowest final weight. The fish in this group also consumed a lot of food without improving their weight and had a high FCR. This may be due to the phytochemical compound in papaya leaf extract that may have a role in intestinal nutrient absorption. Papaya leaf extract contains polyphenols, saponin, flavonoids, and tannins (Saeed et al., 2014). Tannins, for example, are phytochemical substances that may modify nutrition absorption in the intestine. A study in grass carp found that 1.25% tannins may reduce intestinal absorptions because tannins impeded nutrient metabolism; thus, grass carp increased their feed intake to acquire appropriate nutrition, which was supported by a high FCR (Yao et al., 2020). It has a possibility that the inclusion level of papaya leaf extract in the diet may be a dose-limiting supplement and, at a rate of 4%, may not be advantageous for red hybrid tilapia.

In addition to the phytochemical compounds, papaya leaf extract contains various proteolytic enzymes that may play a role and alter its efficacy as a feed supplement. Papain is one of four cysteine proteases found in papaya leaf extract, accounting for <10% of the total cysteine proteases found (Saeed et al., 2014). Other compounds in the papaya leaf that can contribute to improved nutrient efficiency and absorption, in addition to papain, include chymopapain A and B, caricain, and glycyl endopeptidase III and IV (Saeed et al., 2014). These proteolytic enzymes work in concert to convert protein to peptones, which are more easily absorbed into the bloodstream, facilitating protein digestion and growth (Goda et al., 2012). Exogenous proteolytic enzymes digest most protein substrates more efficiently than pancreatic proteolytic enzymes. Papain, for instance, has broad binding affinity, splitting peptide bonds of basic amino acids, leucine, or glycin as well as hydrolyses esters and amides (Vij and Prashar, 2015, Mahanty et al., 2013, Goda et al., 2012).

Although at 4% inclusion of papaya leaf extract stunted growth, the inclusion of papaya leaf extracts up to 4% showed no significant influence on blood parameters and the biochemical composition of red hybrid tilapia except for serum urea, suggesting that the inclusion of papaya leaf extract does not alter the normal blood conditions. Besides, the inclusion of papaya leaf extract reduces serum urea in red hybrid tilapia. Serum urea is an indicator of renal and liver function. An elevation in BUN level signifies hepatotoxicity and nephrotoxicity (Yao et al., 2020, Kantham, 2009). The protective effects of *Carica papaya* seed extract against nephrotoxicity (Madinah et al., 2015) and *Carica papaya* extract against hepatotoxicity (Kantham, 2009) in rats were consistent with the serum urea trend seen in this study.

## 5 Conclusion

In conclusion, papaya leaf extract has the potential to be used as an aquafeed additive at 1% or 2% inclusion levels in aquafeed to promote better growth performance in red hybrid tilapia without compromising regular digestive enzyme activity blood parameters and biochemistry, or the fish's proximate composition. However, further studies of papaya leaf compounds on fish, nutrient absorption, and the mechanism of action by these compounds may provide more knowledge, particularly in producing organic agricultural by-product aquafeed additives to support sustainable aquafeed.

## CRediT authorship contribution statement

**Noor Khalidah Abdul Hamid:** Writing – original draft, Conceptualization, Methodology. **Peace Onas Somdare:** Data curation, Software, Formal analysis. **Khadijah Abdullah Md Harashid:** Data curation, Software, Formal analysis. **Nurul Ain Othman:** Data curation, Software, Formal analysis. **Zulhisyam Abdul Kari:** Writing – review & editing. **Lee Seong Wei:** Data curation, Software, Formal analysis. **Mahmoud A.O. Dawood:** Writing – review & editing.

## Declaration of Competing Interest

The authors declare that they have no known competing financial interests or personal relationships that could have appeared to influence the work reported in this paper.
